# A spectral reflectance-based approach for formulating color mixing recipes for single- and multi-layered woven structures

**DOI:** 10.1038/s41598-022-19578-7

**Published:** 2022-09-08

**Authors:** Youngjoo Chae

**Affiliations:** Department of Clothing & Textiles, Chungbuk National University, Chungdae-ro 1, Seowon-gu, Cheongju, Chungbuk 28644 Republic of Korea

**Keywords:** Materials science, Physics

## Abstract

The color of yarn dyed woven fabrics comes from a series of different colored yarns mixed in complex ways and various proportions. Thus, predicting the color mixing effect or formulating the recipe is a difficult task which should consider the interaction between colored yarns and structure variations. Color mixing recipes, which are also called color prediction models, for woven fabrics have generally been derived through the two-dimensional modeling of woven structures. In this study, the three-dimensional geometrical and colorimetrical modelings of single-, double-, and three-layered woven fabrics in a wide range of colors were conducted to obtain two types of optimal spectral reflectance-based color prediction models. Through model evaluations, the obtained three-dimensional color prediction models were proved to have much higher predictive accuracy, especially in terms of lightness and chroma predictions, than that of the two-dimensional color prediction models previously developed.

## Introduction

Computer-aided design (CAD) has been a huge revolution in the textile industry. Thanks to CAD systems, textile designers can now visualize their imaginary designs with a great speed and ease at the click of a mouse on a computer screen and check the designs in the final forms of fabric before actual production. By checking the simulated fabrics, designers can easily modify their designs if needed by reassigning weaves and yarn colors on a computer screen. For these reasons, the need for physical sampling, a very time-consuming and costly process to match the final fabric to the original design, has been reduced significantly when compared to the traditional, non-computerized fabric design method. However, there still is a discrepancy between the actual and the simulated fabrics, especially in terms of color.

**Figure 1:**
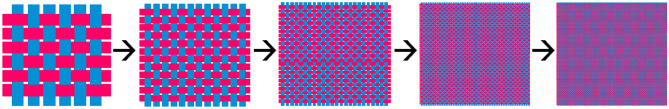
Optical color mixing in a woven structure.

The color of yarn dyed woven fabrics comes from a series of different colored yarns interlaced in complex ways and various proportions. Thus, predicting or reproducing the color is a difficult task that requires pertinent color mixing recipes. The color mixing recipes for yarn dyed woven fabrics should be different from those for colors in other media, such as painting, printing, monitor, etc., because different types of colors are governed by different color mixing principles. There are three typical color mixing principles: the additive color mixing of lights, the subtractive color mixing of colorants, and optical color mixing^1^. Basically, additive and subtractive color mixings are physical phenomena in which an equal mixture of three primaries, red, green, and blue for lights and cyan, magenta, and yellow for colorants, produces white and black, respectively. However, optical color mixing is a psychophysiological phenomenon, that is, color illusion caused when two or more colors are arranged in juxtaposition like in yarn dyed woven fabrics. When the different colored yarns juxtaposed in a woven structure are observed from a distance, the colors are optically mixed and perceived as a single color^2^. For example, the color of the fabric woven with red and blue yarns may be perceived as purple as illustrated in Fig. 1. Therefore, there should be color mixing recipes for yarn dyed woven fabrics that can predict and reproduce the optical color mixing effects accurately with the consideration of complex fabric structures. Provided that the accurate color mixing recipes are applied to current CAD systems, the simulations of the final fabrics to be produced will present the colors more closely akin to those of actual fabrics, probably resulting in the elimination of physical sampling. However, comparatively little research has been conducted to develop color mixing recipes, i.e. color prediction models, for yarn dyed woven fabrics.

In the works of Dimitrovski and Gabrijelčič (or Gabrijelčič and Dimitrovski)^3–5^, L*a*b*-based linear color prediction model (D-G model), by which the color attributes of complex woven structures can be predicted relatively easily from the lightness L*, redness-greenness a*, and yellowness-blueness b* values of the yarns used, was developed. Mathur^6^, Mathur et al.^7^, and Seyam and Mathur^8^ have evaluated the accuracy of the D-G model and the other two color prediction models previously developed for other types of colors when applied to woven fabrics. Chae^2^ and Chae et al.^9,10^ have conducted the numerical optimization of eight types of existing color prediction models for better predictive accuracy. In another study by Chae et al.^11^, new color prediction models were derived for single- and double-layered woven fabrics by considering their layered structures.

The previous studies have provided adequate theoretical bases for developing new color prediction models for woven structures. However, most of the previous models yielded unsatisfactory color prediction results, especially in terms of lightness predictions. There could be two reasons for this: the first is the minimal modification of the previous color prediction models developed for other types of colors, such as dyed, painted, and printed colors; the second is the two-dimensional modeling of three-dimensional woven structures. With respect to two-dimensional modeling, most of the previous models postulate that woven fabrics have two-dimensional flat structures when calculating the proportion of each yarn color on the fabric surface. This postulation neglects the shadows caused by height variation at yarn intersections that affect not only the lightness of the fabric but also the chroma; thus, this leads to inaccuracies in overall color predictions. Although Chae et al.^11^ have conducted the three-dimensional structural modeling of woven fabrics, the resultant color prediction models are applied only to single- and double-layered fabrics.

The ultimate goal of this study is to suggest optimal color prediction models not only for single-layered woven fabrics, but also for multi-layered woven fabrics composed of multiple colors of yarns. The specific objectives are as follows: (1) to demonstrate the unsuitability of previous two-dimensional color prediction models for three-dimensional woven structures by comparing the actual color attributes of single-, double-, and three-layered woven fabrics in the CIELAB color space; (2) to numerically verify the general inaccuracy of two-dimensional color prediction models by calculating the differences between the predicted and the actual colors of fabrics; and (3) to develop optimal color prediction models, that is, three-dimensional color prediction models, with improved accuracy through the geometrical and colorimetrical optimizations of two-dimensional modeling.

## Methodology

### Samples

A total of 210 single-, double-, and three-layered woven fabrics in a variety of colors were designed and produced using the Arahne CAD system and the Staubli LX 3202 jacquard machine. White yarns were used for warp, and four primary colors of red, yellow, green, and blue yarns were used for weft in various proportions. All the yarns were the same polyester filament yarn with a diameter of 0.175 mm. In the case of double- and three-layered samples, all layers in the sample were produced to have the same color, that is, weft color arrangement, and the same weave, that is, 1/3 twill or 1/7 sateen, with a yarn density (warp × weft/cm) of 39 × 30/cm and 45 × 30/cm, respectively. To stitch the layers together in the samples, a self-stitching method, in which some warps in the face layer stitch down the other layers, was used so that the face color of the sample was not affected. The weft color arrangements and weaves applied identically to all layers in double- and three-layered samples were also applied to single-layered samples. Table 1 shows the color and weave designs of the 210 woven fabric samples produced.

**Table 1:**
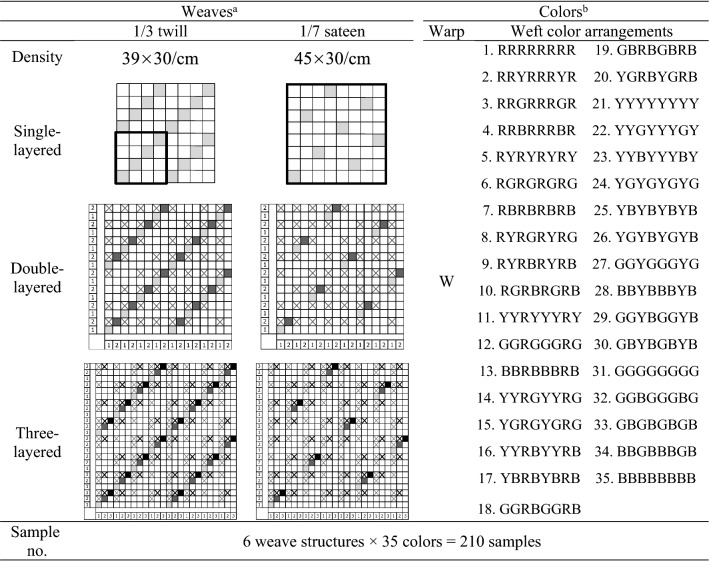
Designs of single-, double-, and three-layered woven fabric samples.

^a^Weaves.

In the images of single-layered samples, a bold box indicates a weave repeat.

In the images of double- and three-layered samples, the mark X indicates a stitching point.

^b^Colors.

The warp color, white, and 35 weft color arrangements were used for single-layered samples and all layers in double- and three-layered samples identically.

W = white yarn; R = red yarn; Y = yellow yarn; G = green yarn; B = blue yarn.

### Color measurement

The spectral reflectance values *R* of 210 fabric samples, the five yarns used to produce the fabric samples, which are white, red, yellow, green, and blue yarns, and the white tile used as a background for fabric sample measurements were measured from 360 to 700 nm with a 10 nm interval by a Datacolor 650 spectrophotometer. When measuring yarns, the yarns were evenly wound onto a neutral matte gray cardboard in six layers without gaps using a custom-made yarn winding machine. From the obtained *R* values, the CIE lightness L*_10_, redness-greenness a*_10_, yellowness-blueness b*_10_, chroma C*_ab,10_, and hue *h*_ab,10_ values were calculated based on the CIE standard illuminant D65 and the CIE 10° standard observer.

### Color prediction modeling

**Figure 2:**
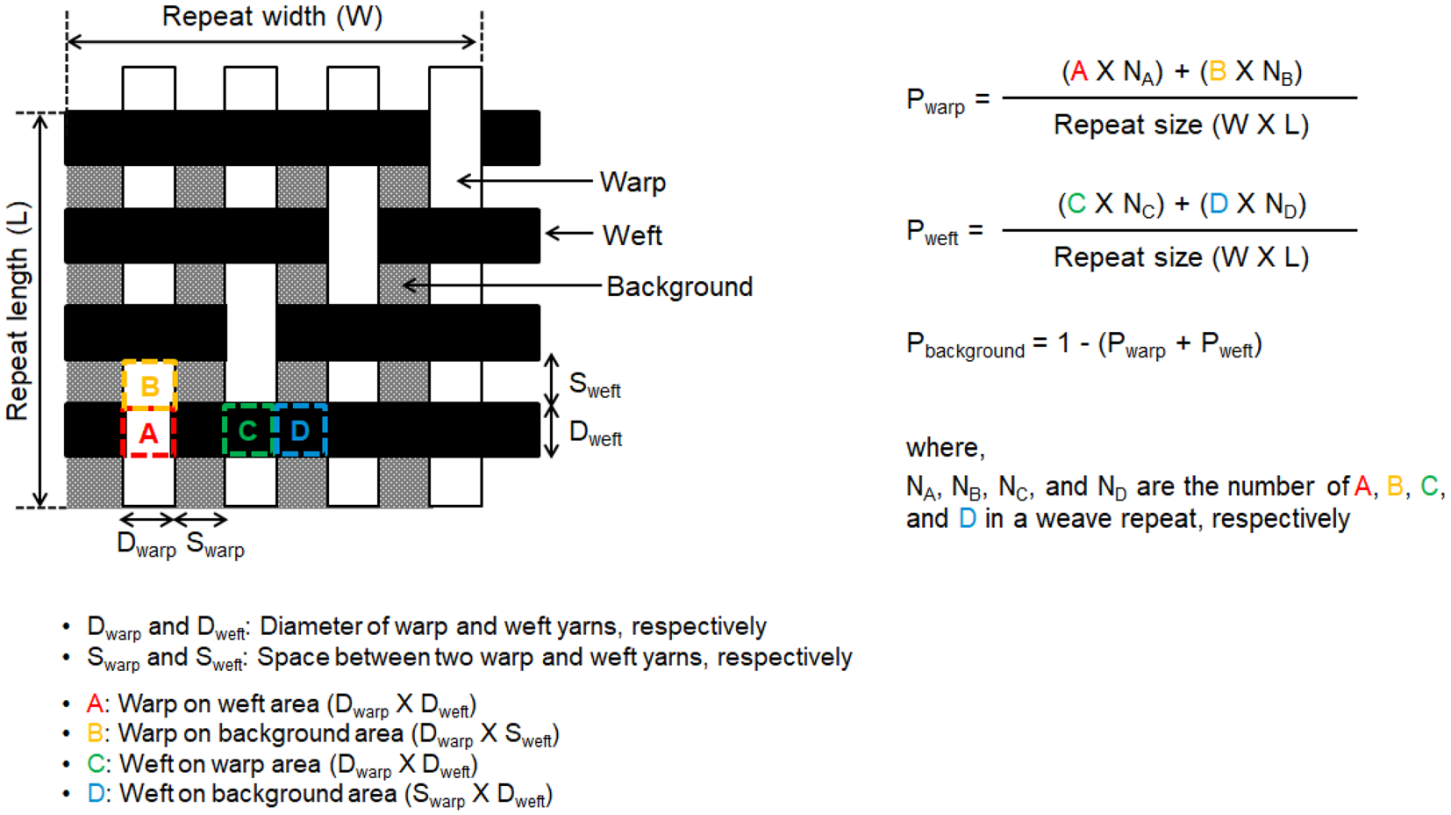
Principle of two-dimensional geometrical modeling of woven fabrics.

The color prediction modeling of yarn dyed woven fabrics is conducted in two steps: geometrical modeling and then colorimetrical modeling. The first step, geometrical modeling, is to calculate the proportion of each yarn color on the fabric surface. As mentioned earlier, two-dimensional modeling is based on the postulation that woven fabrics have two-dimensional flat structures and has usually been carried out in order to simplify the calculation of each color proportion in complex three-dimensional woven structures. The fundamental principle of two-dimensional geometrical modeling is shown in Fig. 2 taking simple 4-thread twill as an example in which the surface consists of three parts: warp color, weft color, and background color. In Fig. 2, P_warp_, P_weft_, and P_background_ refer to the proportion of warp color, weft color, and background color, respectively. The P_warp_ and P_weft_ are calculated by dividing the area covered by the relevant color by the total surface area in the weave repeat. Since the sum of all color proportions is 1 (100%), P_background_ can be calculated by subtracting P_warp_ and P_weft_ from 1.

The next step, colorimetrical modeling, is to predict the final color attributes L*_10_, a*_10_, b*_10_, C*_ab,10_, and *h*_ab,10_ of woven fabrics from the calculated proportions P and the measured color attributes of yarns and background by using color prediction models. The Eqs. (1)–(6) are the four color prediction models that have been applied to woven fabrics: log *K/S* model (*K/S*-based linear model, Eq. (1))^12^; Viggiano model (V model: *R*-based linear model, Eq. (2))^13^; Warburton-Oliver model (W-O model: *R*-based exponential model, Eq. (3))^14^; and Dimitrovski- Gabrijelčič model (D-G model: L*a*b* (previous notation of L*_10_a*_10_b*_10_)-based linear model, Eqs. (4)–(6))^3–5^.

1 $${\text{log}}\left( {K/S} \right)_{{{\text{mixture}}}} = \mathop \sum \limits_{i = 1}^{n} {\text{P}}_{i} \cdot{\text{log}}\left( {K/S} \right)_{i}$$

2 $$R_{{{\text{mixture}}}} = \mathop \sum \limits_{i = 1}^{n} {\text{P}}_{i} \cdot R_{i}$$

3 $$R_{{{\text{mixture}}}} = \mathop \prod \limits_{i = 1}^{n} R_{i}^{{{\text{P}}_{i} }}$$

4 $$L^{*}_{{{\text{mixture}}}} = \mathop \sum \limits_{i = 1}^{n} {\text{P}}_{i} \cdot L^{*}_{i}$$

5 $$a^{*}_{{{\text{mixture}}}} = \mathop \sum \limits_{i = 1}^{n} {\text{P}}_{i} \cdot a^{*}_{i}$$

6 $$b^{*}_{{{\text{mixture}}}} = \mathop \sum \limits_{i = 1}^{n} {\text{P}}_{i} \cdot b^{*}_{i}$$

where P_*i*_ refers to the two-dimensionally calculated proportion of the color *i* in the mixture (a value between 0 and 1), *K/S*_*i*_ refers to the *K/S* value (absorption coefficient/scattering coefficient) of the color *i*, *R*_*i*_ refers to the spectral reflectance of the color *i* (a value between 0 and 1), and L*_i_ a*_i_ b*_i_ refer to the CIE lightness, redness-greenness, and yellowness-blueness values of the color *i*.

**Figure 3:**
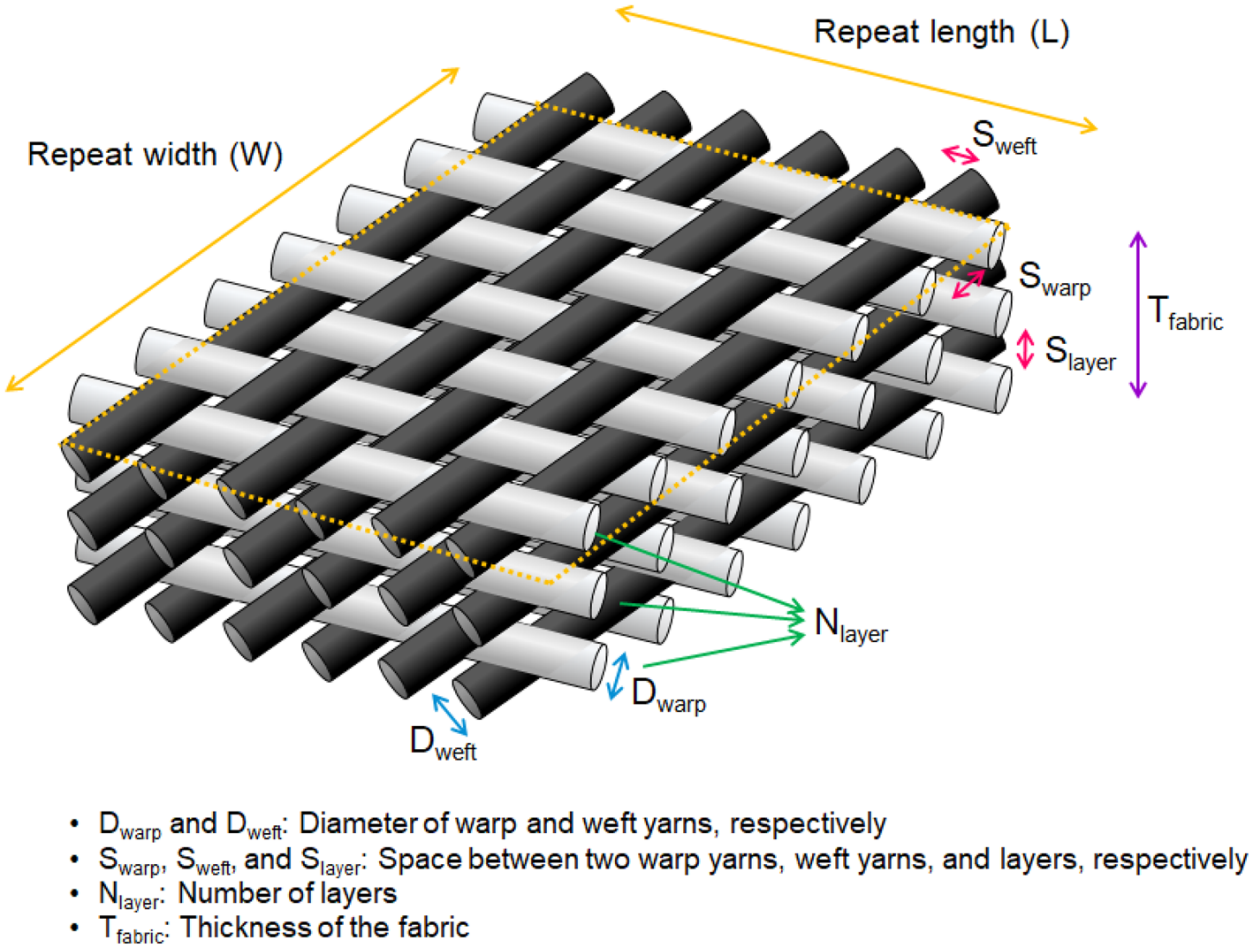
Three-dimensional structural parameters of single- and multi-layered woven fabrics.

The above two-dimensional color prediction modeling fails to consider not only height variation at yarn intersections, but also lower layers in double- or multi-layered fabrics; thus, all fabrics with the same face layer are predicted to have the same color attributes. To avoid the insufficiencies inherent in two-dimensional color prediction modeling, the geometrical and colorimetrical optimizations of two-dimensional modeling, i.e., three-dimensional color prediction modeling, was conducted in this study. The three-dimensional color prediction modeling of single- and multi-layered yarn dyed woven fabrics is based on the following four major postulations: (1) yarns are uniform cylinders with consistent thickness; (2) when yarns are layered or intersect, the thickness of the yarns remains unchanged, and there are no gaps between the yarns in fabric height direction; (3) yarns are evenly dyed; and (4) lower layers in double- or multi-layered fabrics affect the color attributes of upper layers through the gaps (background areas in Fig. 2) in upper layers. Figure 3 shows the key three-dimensional structural parameters of single- and multi-layered woven fabrics including yarn thickness, yarn arrangement, fabric density, fabric thickness, and the number of layers. By taking these structural parameters into consideration, two types of spectral reflectance-based three-dimensional color prediction models were developed: *R*-based linear model (optimized V model) and *R*-based exponential model (optimized W-O model). The reason for developing the models based on spectral reflectance *R* is that the *R* of yarns has been reported to be a better predictor than the other colorimetric attributes of yarns, such as XYZ tristimulus values, K/S, and L*_10_ a*_10_ b*_10_, for the color predictions of woven fabrics^10,11^. The three-dimensional color prediction models are given in the *Development of three-dimensional color prediction models* section.

Half (35 single-layered sateen samples, 35 double-layered twill samples, and 35 three-layered sateen samples) of 210 single-, double-, and three-layered samples were used to develop three-dimensional color prediction models, and the other half (35 single-layered twill samples, 35 double-layered sateen samples, and 35 three-layered twill samples) were used as test samples to evaluate the accuracy of the models. For the accuracy evaluation, first, the L*_10_, a*_10_, b*_10_, C*_ab,10_, and *h*_ab,10_ values of test samples were predicted by using three-dimensional color prediction models. Then, the color differences, that is, lightness difference ΔL*_10_, chroma difference ΔC*_ab,10_, absolute hue difference **│**Δ*h*_ab,10_│, and overall color difference Δ*E*_CMC(2:1)_, between the predicted and the measured colors of test samples were calculated as error values. The reason for using absolute values for hue difference, unlike lightness and chroma differences, is that discussing whether Δ*h*_ab,10_ is a positive or negative value could be meaningless since *h*_ab,10_ includes four different subattributes: the degrees of redness-yellowness (0 ~ 90), yellowness-greenness (90 ~ 180), greenness-blueness (180 ~ 270), and blueness-redness (270 ~ 360). Finally, the error values of three-dimensional color prediction models were compared with those of two-dimensional color prediction models.

## Results and discussion

### Color differences between single-, double-, and three-layered woven fabrics

**Figure 4:**
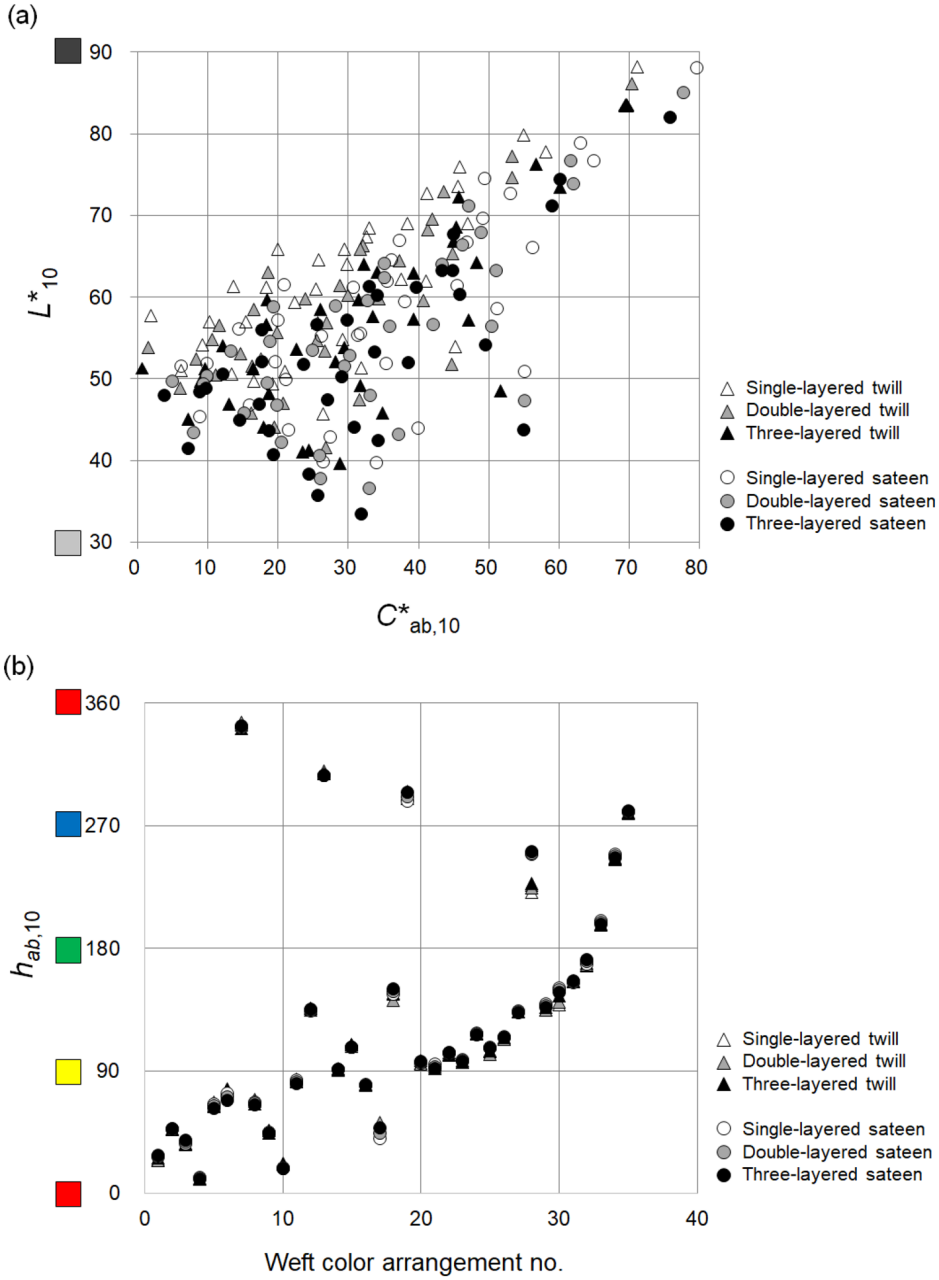
Distribution of single-, double-, and three-layered fabric samples in the CIELAB color space: (**a**) L*_10_C*_ab,10_; (**b**) *h*_ab,10_ spaces.

As shown in Fig. 4, 210 single-, double-, and three-layered fabric samples were distributed in the CIELAB color space based on their instrumentally measured color attributes. In the distribution, white triangles and circles indicate single-layered twill and sateen samples, respectively, and gray and black ones indicate double- and three-layered samples, respectively. As stated earlier, two-dimensional color prediction models fail to consider the effect of lower layers on the color of the face layer in double- and multi-layered fabrics through gaps between yarns—that is, the models consider the lower layers to be completely concealed beneath the face layer. Thus, two-dimensional color prediction models predict all the single-, double-, and three-layered samples with the same face layer, like the samples used in this study, to have exactly the same color attributes. However, the distribution of samples in Fig. 4 indicates that single-, double-, and three-layered samples actually have different color attributes: as the number of layers in samples increases, the color of the samples becomes darker and less saturated (Fig. 4a). The average lightness differences between single- and double-layered samples and between double- and three-layered samples were − 2.86 ΔL*_10_ (Double L*_10_ − Single L*_10_) and − 2.48 ΔL*_10_ (Three L*_10_ − Double L*_10_), respectively, and the average chroma differences were − 1.45 ΔC*_ab,10_ (Double C*_ab,10_ − Single C*_ab,10_) and − 1.11 ΔC*_ab,10_ (Three C*_ab,10_ − Double C*_ab,10_), respectively. As for hue differences (Fig. 4b), the average differences between single- and double-layered samples and between double- and three-layered samples were 1.09 **│**Δ*h*_ab,10_│ (**│**Double *h*_ab,10_ − Single *h*_ab,10_**│**) and 1.20 **│**Δ*h*_ab,10_│ (**│**Three *h*_ab,10_ − Double *h*_ab,10_**│**), respectively. These results justify the need for three-dimensional color prediction models to predict the colors of single-, double-, and three-layered woven fabrics differently.

### Inaccuracy of two-dimensional color prediction models

**Figure 5:**
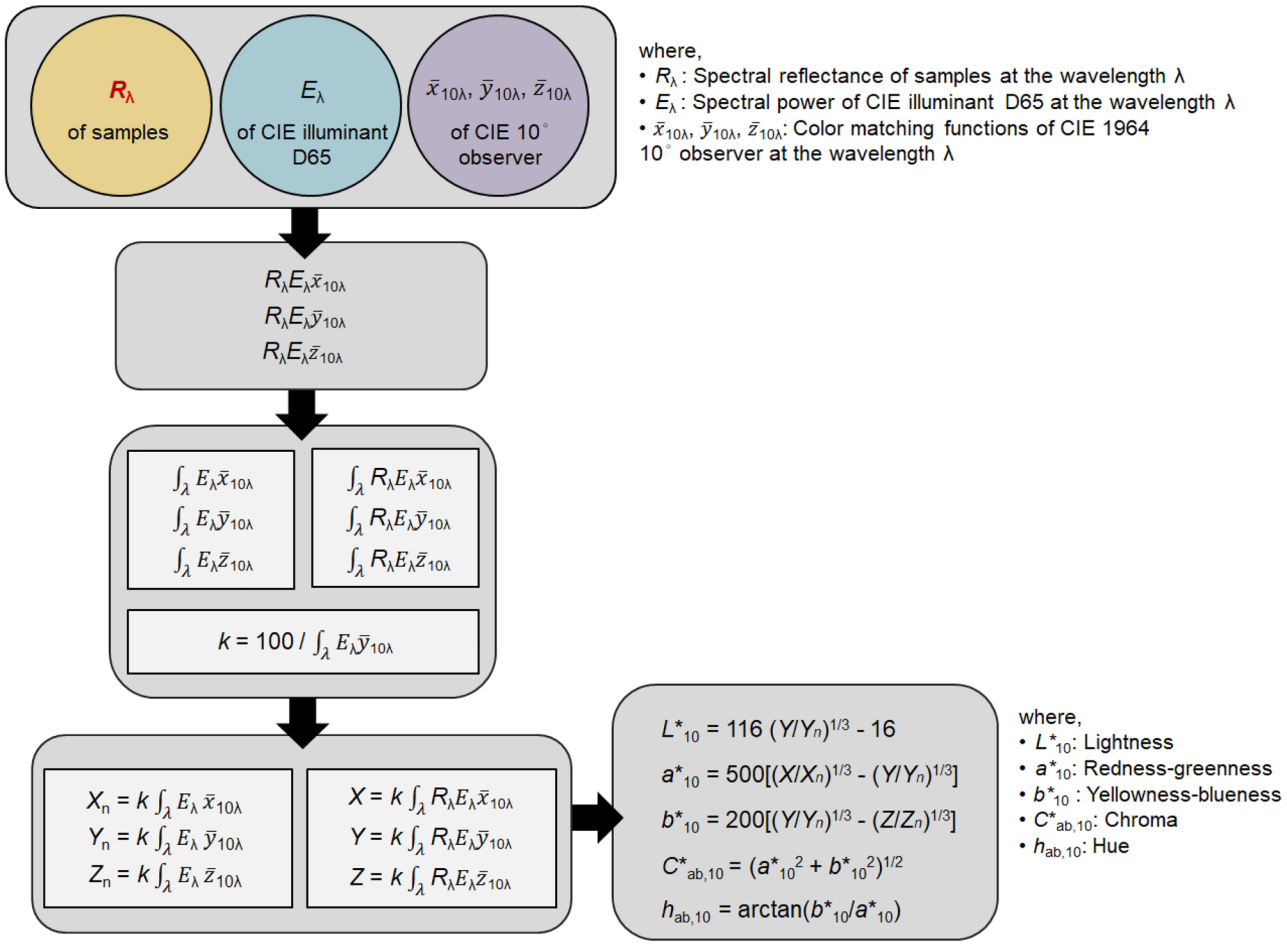
Procedure for calculating L*_10_, a*_10_, b*_10_, C*_ab,10_, and *h*_ab,10_ values from *R* values.

The spectral reflectance values *R* of 105 test samples, that is, 35 single-layered twill fabrics, 35 double-layered sateen fabrics, and 35 three-layered twill fabrics, were predicted from the measured reflectance *R* values of yarns, that is, white, red, yellow, green, and blue yarns, and the background and their proportions on the fabric surface using two types of two-dimensional color prediction models, V model Eq. (2) and W-O model Eq. (3), respectively. The proportion of each yarn and background was obtained through the two-dimensional geometrical calculations presented in Fig. 2. The predicted *R* values of test samples were then converted into L*_10_, a*_10_, b*_10_, C*_ab,10_, and *h*_ab,10_ values based on the spectral power distribution of CIE illuminant D65^15^ and CIE 10° color matching functions^15^ according to the colorimetric calculation procedure suggested by CIE^16^. Figure 5 schematically illustrates how the L*_10_, a*_10_, b*_10_, C*_ab,10_, and *h*_ab,10_ values of test samples were obtained from their *R* values.

**Figure 6:**
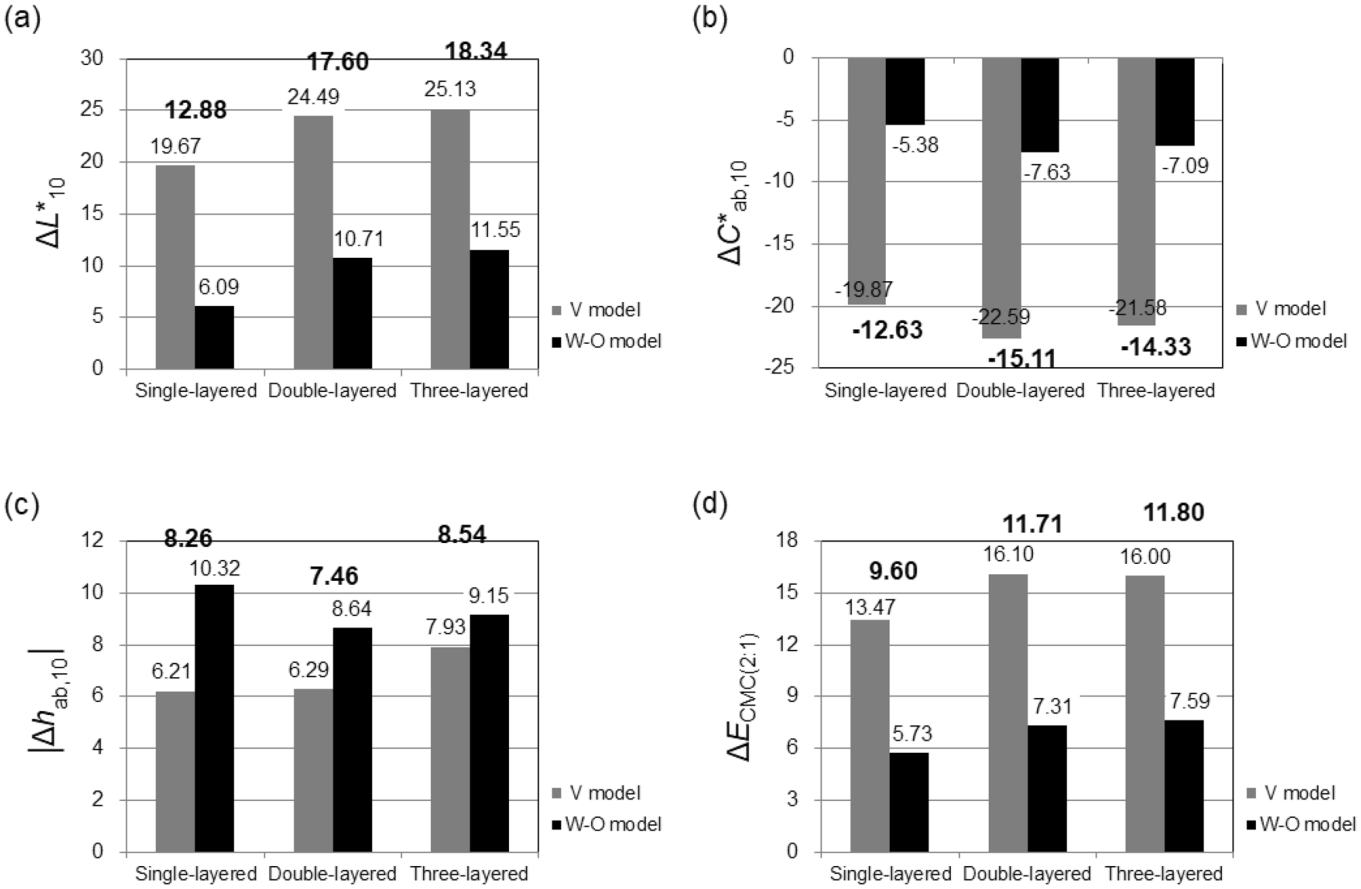
Average color differences, (**a**) ΔL*_10_, (**b**) ΔC*_ab,10_, (**c**) │Δ*h*_ab,10_│, and (**d**) Δ*E*_CMC(2:1)_, between the measured and the predicted colors of single-, double-, and three-layered fabric samples yielded by two-dimensional color prediction models. The color differences were calculated by subtracting the measured color attribute from the predicted color attribute.

In order to demonstrate the inaccuracy of two-dimensional color prediction models numerically, the color differences ΔL*_10_, ΔC*_ab,10_, **│**Δ*h*_ab,10_│, and Δ*E*_CMC(2:1)_ between the measured and the predicted colors of test samples were calculated as error values. The reason for using absolute values for hue difference has been discussed earlier. Taking two test samples, single- and three-layered samples with the same face layer, as an example, Table 2 compares their measured and predicted color attributes and shows the resultant overall color prediction error Δ*E*_CMC(2:1)_. Although the two samples had the same face layer (with the same weave, i.e., 1/3 twill, and the same color, i.e., the weft color arrangement 10, as shown in Table 1), their measured color attributes were different. However, their two-dimensionally predicted color attributes were the same. The ΔL*_10_, ΔC*_ab,10_, **│**Δ*h*_ab,10_│, and Δ*E*_CMC(2:1)_ between the measured and the predicted colors of 105 test samples were averaged for single-, double-, and three-layered samples, separately, and the results are given in Fig. 6.

**Table 2:**
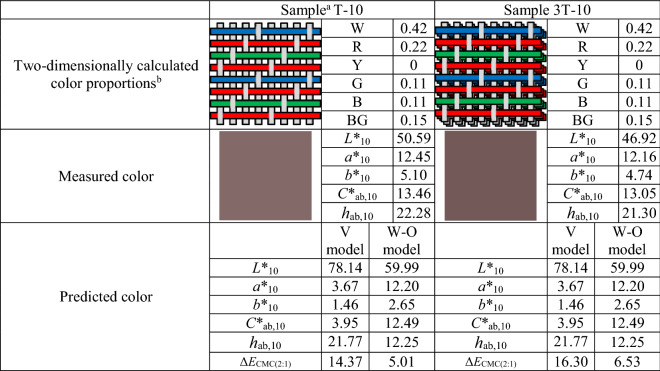
Comparison of color measurement results and two-dimensional color prediction results for two test samples with the same face layer.

^a^Sample.

Weave (T: 1/3 twill)—Weft color arrangement (see Table 1; 10: RGRBRGRB).

^b^Two-dimensionally calculated color proportions.

W = white yarn (warp); R = red yarn (weft 1); Y = yellow yarn (weft 2); G = green yarn (weft 3); B = blue yarn (weft 4); BG = background (white tile).

In general, the colors of double- and three-layered samples were predicted more inaccurately than those of single-layered samples by two-dimensional color prediction models. In lightness predictions, the predicted colors of double- and three-layered samples were lighter than those of single-layered samples, which is contrary to the result of color measurements discussed in the previous section. Additionally, regardless of the number of layers in samples, it is obvious that both two-dimensional color prediction models yielded high error values in all lightness, chroma, hue, and overall color predictions for all types of samples. In particular, V model showed much larger inaccuracy in lightness, chroma, and overall color predictions than W-O model.

As for the error values in overall color predictions of individual models, the ranges of Δ*E*_CMC(2:1)_ of V model and W-O model were from 10 to 24 (average: 15.19) units and from 3 to 13 (average: 6.88) units, respectively, which were high, actually extremely high in the case of V model, compared to the industrial color tolerance standards. In the case of the errors in lightness and chroma predictions, both models predicted the colors of all 105 samples to be much lighter and much desaturated than the actual measured colors with the average error values of 16.27 (V model: 23.09; W-O model: 9.45) ΔL*_10_ (predicted L*_10_ − measured L*_10_) and − 14.02 (V model: − 21.34; W-O model: − 6.70) ΔC*_ab,10_ (predicted C*_ab,10_ − measured C* _ab,10_). The trend of lightness prediction results, that is, predicted colors were much lighter than the actual colors, agrees with previous studies^2,6–11^ on the color prediction of woven structures. It has been believed that the much higher lightness values of predicted colors are due to the color predictions conducted based on the two-dimensional structural modeling of woven fabrics without considering the shadows caused by curved yarns at weave intersections, and this results in inaccuracies not only in lightness predictions but also in chroma predictions. In hue predictions, the average error values of V model and W-O model, 6.81 and 9.37**│**Δ*h*_ab,10_│ units, respectively, indicate the inaccuracy of the models in hue predictions as well. Unlike the results of lightness and chroma predictions shown in Fig. 6a and b, in which the error values of W-O model are much lower than those of V model, W-O model was found to be more inaccurate in hue predictions as can be seen in Fig. 6c.

### Development of spectral reflectance-based three-dimensional color prediction models

Two types of three-dimensional color prediction models, spectral reflectance *R*-based linear model and exponential model, were developed through the geometrical and colorimetrical optimizations of two-dimensional color prediction models, V model and W-O model, respectively. In the three-dimensional color prediction models, the three-dimensional structural parameters of woven fabrics, including yarn thickness, yarn arrangement, fabric density, fabric thickness, and the number of layers, are included as key predictors. The three-dimensional color prediction models are given in Eqs. (7) and (8).

Three-dimensional *R*-based linear model (Optimized V model):

7 $$R_{{{\text{fabric}}}} = \sum\limits_{i = 1}^{n} {\left\{ {{\text{o}}_{i} \cdot {\text{P}}_{i} {\text{ N}}({\text{D}}_{i} + 1) \cdot R_{i} } \right\} + \left( {{\text{o}}_{BG} \cdot {\text{P}}_{BG} \cdot R_{BG} } \right) + {\text{C}}}$$

Three-dimensional *R*-based exponential model (Optimized W–O model):

8 $$R_{{{\text{fabric}}}} = \mathop \prod \limits_{i = 1}^{n} \left\{ {R_{i}^{{{\text{o}}_{i } \cdot {\text{ P}}_{i} {\text{ N}}({\text{D}}_{i} + 1)}} } \right\} \cdot \left( {R_{BG}^{{{\text{o}}_{BG } \cdot {\text{ P}}_{BG} }} } \right) + {\text{C}}$$

where subscripts *i* and *BG* refer to yarn colors (*W*: white; *R*: red; *Y*: yellow; *G*: green; *B*: blue) and background color, respectively, o refers to an optimized coefficient for the relevant color which was obtained by linear regression for linear model or Poisson regression for exponential model, P refers to the proportion of the relevant color in the fabric (a value between 0 and 1), N refers to the number of layers in the fabric, D refers to the diameter of the relevant colored yarn in millimeters (mm), *R* refers to the spectral reflectance of the relevant color (a value between 0 and 1), and C is a constant.

When developing the three-dimensional color prediction models, the number of layers N and the yarn thickness D were taken into account only for yarn colors since background is a two-dimensional color component. In the models, the reason for adding 1 to the yarn thickness D is that the value by which yarn colors are weighted could be below 1 (0.175 mm in this study). The optimized coefficients o for color components and the constant C were obtained based on the calculated P and the measured reflectance *R* values of half of the 210 samples produced, excluding test samples. The optimized coefficients and constant obtained are presented in Table 3.

**Table 3:** Optimized coefficients and constant in three-dimensional color prediction models.

	Coefficients^c^						Constant
	o_*W*_	o_*R*_	o_*Y*_	o_*G*_	o_*B*_	o_*BG*_	C
*R*-based linear model^a^	− 0.3911	0.6218	0.6004	0.6765	0.6927	− 0.0053	0.3826
*R*-based exponential model^b^	0.6470	0.5307	0.5916	0.5993	0.5371	1.2828	0

^a^*R*-based linear model: see Eq. (7).

^b^*R*-based exponential model: see Eq. (8).

^c^Coefficients: o_*W*_ for white yarn; o_*R*_ for red yarn; o_*Y*_ for yellow yarn; o_*G*_ for green yarn; o_*B*_ for blue yarn; o_*BG*_ for background (white tile).

**Figure 7:**
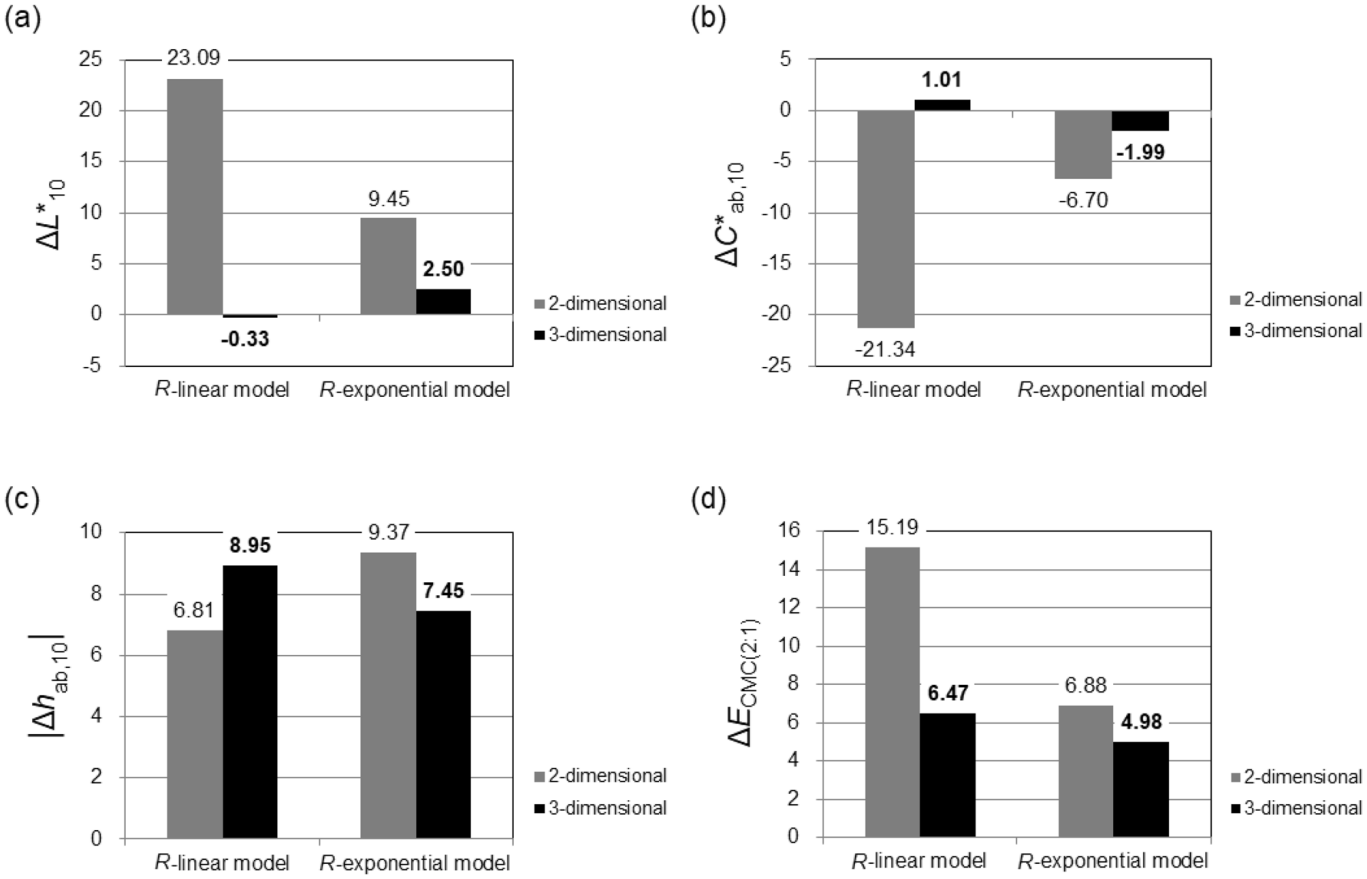
Comparison of average error values, (**a**) ΔL*_10_, (**b**) ΔC*_ab,10_, (**c**) │Δ*h*_ab,10_│, and (**d**) Δ*E*_CMC(2:1)_, between two-dimensional and three-dimensional color prediction models. The error values were obtained by subtracting the measured color attribute from the predicted color attribute.

In order to evaluate the accuracy of three-dimensional color prediction models, the average error values, that is, ΔL*_10_, ΔC*_ab,10_, **│**Δ*h*_ab,10_│, and Δ*E*_CMC(2:1)_, between the measured and the newly predicted colors of 105 test samples, were calculated and then compared with those of two-dimensional color prediction models. The results are presented in Fig. 7. As can be seen in Fig. 7d, the accuracy in overall color predictions for woven fabrics has improved after the geometrical and colorimetrical optimizations of two-dimensional color prediction modeling, that is, three-dimensional modeling, with a decrease in average Δ*E*_CMC(2:1)_ from 15.19 to 6.47 (SD: 2.53) units for *R*-based linear model and from 6.88 to 4.98 (SD: 1.97) units for *R*-based exponential model. In particular, in lightness and chroma predictions, a substantial improvement in accuracy has been achieved with decreased average error values from 23.09 to − 0.33 ΔL*_10_ units and from − 21.34 to 1.01 ΔC*_ab,10_ units for *R*-based linear model and from 9.45 to 2.5 ΔL*_10_ units and from − 6.7 to − 1.99 ΔC*_ab,10_ units for *R*-based exponential model (Fig. 7a and b). On the other hand, a relatively small or no improvement in hue predictions has been realized after three-dimensional modeling (Fig. 7c). In the case of *R*-based linear model, the accuracy has decreased rather than increased with an increased average error value from 6.81 to 8.95 **│**Δ*h*_ab,10_│units. The increase of 2 **│**Δ*h*_ab,10_│ units, however, is a relatively negligible increase when considering that *h*_ab,10_ ranges from 0 to 360, while L*_10_ and C*_ab,10_ range from 0 to 100 and from 0 to no limit in both positive and negative directions, respectively. Since three-dimensional color prediction modeling substantially improved the accuracy in lightness and chroma predictions, without improvement in hue predictions, far more accurate overall color predictions have been achieved than those of two-dimensional color prediction modeling.

The results of accuracy evaluations have proved that the three-dimensional spectral reflectance-based color prediction models developed in this study will be able to replace previous two-dimensional color prediction models successfully. The major shortcoming of two-dimensional models, which fails to consider fabric height variations at yarn intersections and lower layers in multi-layered fabrics resulting in inaccuracies in lightness and chroma predictions, has been minimized. Although the average error values ΔL*_10_, ΔC*_ab,10_, **│**Δ*h*_ab,10_│, and Δ*E*_CMC(2:1)_ of three-dimensional color prediction models are not zero, the values are much lower than those of many other previous models ranging up to 31 Δ*E*_CMC(2:1)_ units^11^. It is envisaged that not only will three-dimensional color prediction models provide textile designers with advantageous conditions to create various woven fabrics in desired colors accurately, but they will also be a solid foundation for developing new color prediction models for woven fabrics and, by extension, knitted fabrics.

## Conclusion

In this study, three-dimensional spectral reflectance-based color prediction models for single- and multi-layered woven fabrics were developed through the geometrical and colorimetrical optimizations of two-dimensional color prediction models. Through model evaluations, three-dimensional models were found to be more accurate than two-dimensional models to predict the colors of various three-dimensional woven fabrics. In particular, in lightness and chroma predictions, three-dimensional models had substantially improved predictive accuracy with far lower error values than those of two-dimensional models. The three-dimensional color prediction models have been proved to be of benefit to the creation of various woven fabrics in desired colors accurately with their potential application to CAD systems. In addition, the method of three-dimensional geometrical and colorimetrical modelings would provide a solid foundation for the development of new color prediction models for more complex woven structures.

## Data Availability

The author confirms that the data supporting the findings of this study are available within the article.
